# Renal function in β-thalassemia major patients treated with two different iron-chelation regimes

**DOI:** 10.1186/s12882-021-02630-5

**Published:** 2021-12-20

**Authors:** Osama Tanous, Yossi Azulay, Raphael Halevy, Tal Dujovny, Neta Swartz, Raul Colodner, Ariel Koren, Carina Levin

**Affiliations:** 1 Pediatric Hematology Unit, Emek Medical Center, 21 Yitzhak Rabin St, Afula, Israel; 2 The Ruth and Bruce Rappaport Faculty of Medicine, Technion – Institute of Technology, Haifa, Israel; 3 Pediatric Department B, Emek Medical Center, Afula, Israel; 4 Pediatric Nephrology Unit, Emek Medical Center, Afula, Israel; 5 Laboratory Department, Emek Medical Center, Afula, Israel

**Keywords:** Thalassemia, uNAG, Iron chelation therapy

## Abstract

**Background:**

Renal injury in transfusion dependent β thalassemia patients (TDT) has been attributed to iron overload, chronic anemia and iron-chelation therapy (ICT) toxicity. We studied renal function in TDT patients treated with two different ICT regimes.

**Patients and methods:**

We studied 36 TDT patients: 26 received deferasirox (DFX) and 10 were treated with deferoxamine (DFO) +/− deferiprone (DFP).

**Results:**

Increased uNAG was found in 30% of the DFX group vs. 10% of the DFO+/−DFP group, the mean uNAG level in the DFX group was significantly higher than in the DFO+/−DFP group, (*P* < 0.05). A moderate negative correlation was found between uNAG levels and mean serum ferritin for the prior 10 years (*P* = 0.03), more pronounced for the DFO+/−DFP group. Twenty nine patients had had their renal function evaluated 10 years earlier; eGFR significantly declined in patients switched to DFX (*P* = 0.0093) but not in patients who continued DFO+/−DFP.

**Conclusions:**

A high prevalence of renal tubular damage was observed in our TDT patients, particularly those treated with DFX; uNAG was negatively associated with mean 10-year serum ferritin, suggesting ICT’s involvement in tubular injury. A significant decline in eGFR compared to a decade earlier was observed only in patients currently treated with DFX. Strict follow-up of renal function in TDT patients is warranted.

## Introduction

β-Thalassemia major (β-TM) is a disease of hemoglobin synthesis leading to ineffective erythropoiesis and transfusion-dependent anemia from the first few months of life. Regular blood transfusions and iron chelation therapy (ICT) markedly improve the survival and quality of life of Transfusion Dependent β Thalassemia (TDT) patients [1, 2], but have led to the emergence of previously unrecognized complications, including renal abnormalities [3–6].

Renal tubular dysfunctions reported in TDT patients include low-molecular-weight proteinuria, found in almost all patients, and increased urinary excretion of calcium, phosphorus, magnesium and uric acid [3–5, 7]. N-acetyl-β-D-glucosaminidase (NAG) is a hydrolytic enzyme found in proximal tubular cells, Urinary NAG (uNAG) levels are considered a reliable marker for early renal tubular injury [8, 9] and were found to be elevated in 35 to 60% of TDT patients [5, 6, 8, 10, 11]. Hyperfiltration, mimicking the early changes of diabetic nephropathy, has been noted in several studies on thalassemic patients [6, 7, 12, 13]. Chronic renal hyperfiltration causes increased proteinuria and in the long term, can cause a progressive decline in glomerular filtration rate (GFR) and renal fibrosis [4, 14–16]. Hemolysis, hypercalciuria and hyperuricosuria have been proposed as causes of the nephrolithiasis observed in TDT patients [17]. Hematuria has been observed in 2 to 10% of TDT patients and might be attributed to the increased incidence of nephrolithiasis [3, 7].

Several mechanisms were suggested as a cause or renal damage in TDT patients, among them chronic anemia, hypoxia, hemosiderosis and ICT. Chronic anemia and hypoxia reduce systemic vascular resistance, and increase renal plasma flow, hyperfiltration, tubular cell injury, apoptosis and ultimately are a cause of renal fibrosis and sclerosis [5, 7, 14] Some of these changes have also been observed in thalassemia intermedia patients [18, 19].

Iron overload, including proximal and distal tubule hemosiderin deposits, have been observed in autopsies of TDT patients [20, 21]. The proposed injury mechanism is through mitochondrial stress and reactive oxygen species [22]. Clinical studies have shown a direct correlation between serum ferritin levels and markers of renal tubular toxicity [23, 24], with reversal of tubular defects after ICT [25, 26].

Three iron chelators are commonly used to prevent or reduce iron overload in β-TM: deferoxamine (DFO), a siderophore administered as a subcutaneous infusion, usually for 8–12 h/day, leading to renal excretion of iron; deferiprone (DFP), an oral iron chelator with a renal route of iron elimination [27, 28] and deferasirox (DFX), an oral iron chelator, using the hepatobiliary route to excrete chelated iron.

Direct toxic action on epithelial tubular cells and induction of tubular apoptosis and necrosis constitute the preferred hypothesis explaining chelator-induced renal injury [15, 29, 30] The clinical effect of chelation toxicity is thought to be mild, reversible and non-progressive [31], Several reports of reversible Fanconi syndrome were also published in patients treated with DFX [32–34]. In a large-scale study, DFX was associated with significant hypercalciuria [35]. Large prospective trials examining renal function in TDT patients receiving different types of chelation are scarce. Over-chelation causing relative iron depletion has been proposed as an indirect mechanism of drug related renal toxicity in TDT patients treated with DFX and DFO. It has shown to cause a decrease in GFR and an increase in serum Cr [5].

Anemia, iron overload and ICT coexist in virtually all TDT patients, then, it is hard to isolate and study each contributing factor separately. The goal of this study is to assess glomerular and tubular function in a cohort of 36 pediatric and adult TDT patients, to determine the prevalence of glomerular and tubular renal function abnormalities and to correlate these findings with hematological and iron-overload parameters, and with the type of ICT. Most of the patients in the current study were evaluated in our previous study [6], conducted a decade earlier. New patients born or started on chelation after the previous study have been recruited and the changes in therapy over time were recorded. The results from the two studies were compared.

## Patients and methods

### Study population

The study was conducted in the Pediatric Hematology Unit of Emek Medical Center, in Afula, Israel. Transfusion-dependent β-TM patients were included in the study, children and adults. Patients’ medical history and laboratory data were obtained from their medical files.

The patients were treated under standard protocols for blood transfusions and ICT based on the Israeli clinical guidelines for treating thalassemia patients and the Thalassemia International Federation guidelines [36, 37]. Patients received regular blood transfusions every 2 to 3 weeks. The chelation protocol consisted of one of three possible options: (1) oral DFX at a dose of 25–40 mg/kg day, once daily; (2) subcutaneous infusion (10–12 h) of DFO at a dose of 40–60 mg/kg per day, 6 days a week; (3) DFO at the standard dose in combination with daily oral DFP at a dose of 60–70 mg/kg/day in three divided doses.

## Methods

### Renal function tests

Blood samples and fresh morning urine samples were obtained at regular follow-up visits, before giving the scheduled blood transfusion. Blood samples were immediately examined for complete blood count, serum creatinine (Cr), electrolytes, ferritin, serum iron and transferrin. Urine samples were immediately evaluated for electrolytes, Cr, calcium (Ca), osmolality, protein and hematuria, and stored at − 80 °C for further uNAG evaluation (colorimetric assay, Diazyme Laboratories, San Diego, CA). The uNAG was considered increased if the result was above 12 IU/l. Estimated GFR (eGFR) was calculated according to the Schwartz formula in pediatric patients and the Chronic Kidney Disease Epidemiology Collaboration (CKD-EPI) formula for adults. Fractional excretion of sodium (FENa), fractional excretion of potassium (FEK), Ca/Cr ratio, uric acid excretion (UAE), and tubular phosphorus reabsorption were calculated using standard formulas. The blood and urine tests were performed during regular follow up visits, before scheduled blood transfusion, while the patients were fasting. The tests were postponed if the patient had fever or acute infectious disease. The urine was the first morning urine. The tests were performed ten years after the assessment reported in our previous article in the period between January and March of 2011 [6].

### Iron status estimation

The further values were calculated for the 10 years period before the study, mean ferritin levels, mean volume of blood transfusions (cc/kg per year) and the total amount of iron transfused. Each unit of blood transfused was considered to contain 4 mmol iron [38].

Results of heart and liver iron content assessed by magnetic resonance imaging (T2*MRI) was recorded if performed, closest in time to the point of renal function assessment. Liver iron concentration was measured in milliseconds by calculating T2*. Cardiac iron concentration was estimated as cT2* and was also measured in milliseconds, and its reciprocal cR2* (1000/cT2*) in Hertz.

### Chelation

To better understand the effects of iron chelation on renal function, the patients were divided into two subgroups according to ICT received at the time of the study: (1) DFX, and (2) DFO+/−DFP.

### Comparison to the results of our previous study

We compared the results of the current study with the results of a study performed ten years earlier in the same cohort of patients [6].

### Statistical methods

Comparison of numerical data among the different groups was performed using unpaired two-tailed Student’s *t* test. Comparisons between the previous and current study in the same patients were performed using two-tailed Wilcoxon signed rank test. Pearson’s or Spearman’s correlation test, depending on the normality of the variables, was performed for the renal parameters uNAG, eGFR, and tubular tests with additional parameters (age, Hb, iron overload parameters). *P* < 0.05 was considered significant.

## Results

Patients’ characteristics and laboratory results from the current and previous studies and patient subgroups are summarized in Tables 1 and 2. A total of 36 TDT patients were studied, 18 males (13 in the DFX group and 5 in the DFO+/−DFP group) and 18 females (13 in the DFX group and 5 in the DFO+/−DFP group). Patient age ranged from 5 to 45 years, Mean age 20.92 ± 9.7. The mean age of the patients treated with DFX was slightly younger than for the DFO+/−DFP group. 19.42 ± 9.3 vs 24.8 ± 10, *P* = 0.14. No significant difference in age or sex was found between the two groups. Twenty nine of the patients participated in the previous study, and the other seven patients were new patients born after the first study was conducted.

**Table 1:** Hematological and iron overload parameters of patients with β-Thalassemia Major and their subgroups based on chelation treatment in the current and previous study^6^

Parameter (normal range)	Current study			Previous study
Iron Chelator	DFX *n* = 26	DFO+/−DFP *n* = 10	Total *n* = 36	DFO *n* = 29
Length of current treatment (mean months)	60.4 ± 9.1	101.2 ± 66.1		
Hemoglobin (g/dl) (12–15)	8.3 ± 0.95	8.12 ± 0.88	8.25 ± 0.9	8.9 ± 0.9
Serum ferritin (ng/ml) (22–322)	2597 ± 1669**	4801 ± 1893**	3209 ± 1976	3839 ± 1780
Total amount of iron transfused for prior 10 years (mmol)	1100 ± 105	1388 ± 80	1180 ± 413	1218 ± 391

Data expressed as mean ± SD

*P < 0.05 between chelation-based subgroups of β-thalassemia patients

**P < 0.05 between total patients in the current study and those in the previous study

**Table 2:** Renal function and biochemical data of patients with β-Thalassemia Major and their subgroups based on chelation treatment in the current and previous study

Parameter (normal range)	Current study			Previous study
Chelator	DFX *n* = 26	DFO +/−DFP *n* = 10	Total *n* = 36	DFO *n* = 29
Gender (M:F)	(13:13)	(5:5)	(18:18)	(13:16)
Age	19.4 ± 9.3	24.8 ± 10.2	20.9 ± 9.5	14 ± 8.1
Serum Cr (mg/dl) (0.6–1.1)	0.64 ± 0.11	0.6 ± 0.14	0.63 ± 0.12	0.54 ± 0.09
Serum sodium (meq/l) (135–145)	137.6 ± 1.7	137.1 ± 2.2	137 ± 1.8	139 ± 2
Serum potassium (meq/l) (3.5–5.5)	4.12 ± 0.3	4.17 ± 0.19	4.12 ± 0.2	4.25 ± 4.6
Serum uric acid (mg/dl) (2.5–7.5)	4.3 ± 1.2	4.3 ± 1.2	4.54 ± 1.2	4.3 ± 0.9
GFR (ml/min per 1.73 m^2^) (> 60)	100.9 ± 17	114 ± 22	104.6 ± 19	109.7 ± 23.2
eGFR (ml/min per 1.73 m^2^)	113.2 ± 21	124.8 ± 26	116.4 ± 23	113.5 ± 26
uNAG (IU/l) (< 12)	10.42 ± 6.1*	5.33 ± 2.7*	9.07 ± 5.8	10.76 ± 6.7
Abnormal uNAG	(8/26) 30.7%	(1/10) 10%	(9/36) 25%	(8/29) 27.5%
Patients with ProtU> 150 mg/d or equivalent prot/ Cr ratio	(2/26) 7.7%	(2/10) 20%	(4/36) 11.1%	
Urine Ca/Cr (< 0.14)	0.229 ± 0.2	0.173 ± 0.1	0.21 ± 0.2	0.09 ± 0.1**
Abnormal Urine Ca/Cr	(8/26) 30.7%	(2/10) 20%	(10/36) 27.8%	(2/29) 6.9%
FeNa (%)	0.84 ± 0.245	0.70 ± 0.33	0.8 ± 0.43	0.94 ± 0.61
FeK (%) (4–16)	13.2 ± 8.3	13.6 ± 8.9	13.3 ± 8.3	16 ± 8.3
UAE (mg/dl GFR) (< 0.56)	0.7 ± 0.24	0.56 ± 0.19	0.66 ± 0.23	0.75 ± 0.24
Abnormal UAE	(18/26) 69.2%	(5/10) 50%	(23/36) 63.8%	(21/29) 72.%
TmP/GFR (mg/dl) (3–5)	4.63 ± 0.68	4.75 ± 0.95	4.67 ± 0.75	4.89 ± 1.15
Urine osmolality (mosmol/kg) (50–1200)	678.2 ± 188	705 ± 222	685.7 ± 193	757 ± 170

Data expressed as mean ± SD

*P < 0.05 between chelation-based subgroups of β-thalassemia patients

**P < 0.05 between total patients in the current study and those in the previous study

### Chelation therapy

**Fig 1:**
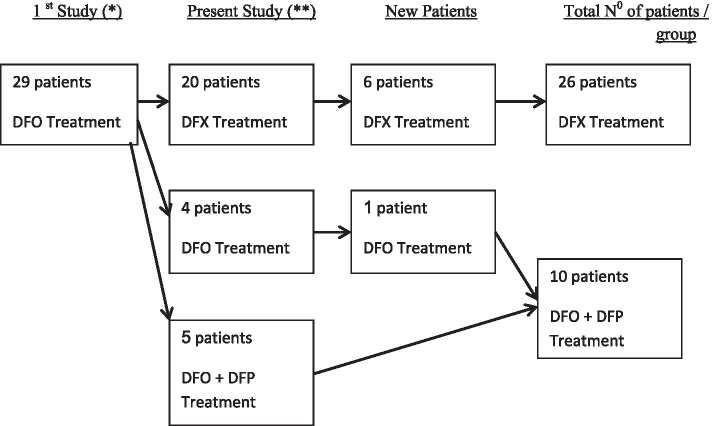
– Patients included in previous study and in the present study according to treatment group. (DFX) Deferasirox. (DFO) Desferrioxamine. (DFP) Deferiprone. (*) Patients included in the previous study [6]. (**) Patients included in previous study and in present study

Patients were divided into two groups based on the ICT they have received during the time of assessment in the present study. The DFX group included twenty six patients and the DFO+/−DFP group included ten patients (Fig 1).

The twenty-six patients received DFX for a mean duration of 59 ± 9.1 months (range 27–70 months). Among them in six patients, DFX was the first and only chelator used. The DFO+/−DFP group included four patients treated with DFO alone for a mean duration of 114.7 ± 64 months (range 47–189) and another six patients treated with DFO and DFP for a mean duration of 21.3 ± 19.7 months (range 3–51).

Twenty patients changed chelation therapy from DFO to DFX after a mean duration of 79.4 ± 34.7 months, nine continued DFO, six of them with the addition of DFP. One patient changed chelation therapy from DFX to DFO+ DFP after 57 months.

### Renal function parameters (Table 2)

Serum Cr, K and Na were within the normal range in all patients, with no significant differences between groups. Abnormal serum uric acid levels were found in five patients: one with high levels in each group, two with low level in the DFX group, and one with lower than normal levels in the DFO + DFP group.

### Tubular function (Table 2)

The mean urinary FeNa was in the normal range for all patients. The mean urinary Ca/Cr ratio was higher, but not statistically significantly, in the DFX group vs. the DFO+/−DFP group. Hypercalciuria (Ca/Cr > 0.25) was found in ten patients (28%): eight patients (30%) in the DFX group and two patients (20%) in the DFO+/−DFP group. None of the patients with hypercalciuria were treated with Ca supplements. No statistically significant difference was found between the two groups.

Increased FeK (> 15%) was found in 12 patients (33%): eight patients (31%) in the DFX group and four patients (40%) in the DFO+/−DFP group. High UAE (> 0.56 mg/dl GFR) was found in both groups; abnormally high levels were present in 23 patients (64%): 18 patients (69%) in the DFX group and five patients (50%) in the DFO+/−DFP group. The renal tubular maximum reabsorption rate of phosphate (TmP/GFR) (normal values 3–5 mg/dl) was elevated in nine patients (25%): six patients (21%) in the DFX group and three patients (30%) in the DFO+/−DFP group. No statistically significant difference was found in all those parameters between the two groups. Most patients (75%) had normal uNAG levels. The mean uNAG levels were significantly higher in the DFX group vs. the DFO+/−DFP group (*P* = 0.012). Abnormally high uNAG values were found in a total of nine patients (25%): 30% in the DFX group vs. 10% in the DFO+/−DFP group (*P* = 0.19).

### Glomerular function

The eGFR was in the normal range for all patients. In the DFX group, eGFR was slightly lower than in the DFO+/−DFP group (*P* = 0.06). **(**Table 2**).**

### Correlation between renal function and parameters of iron overload

**Fig. 2:**
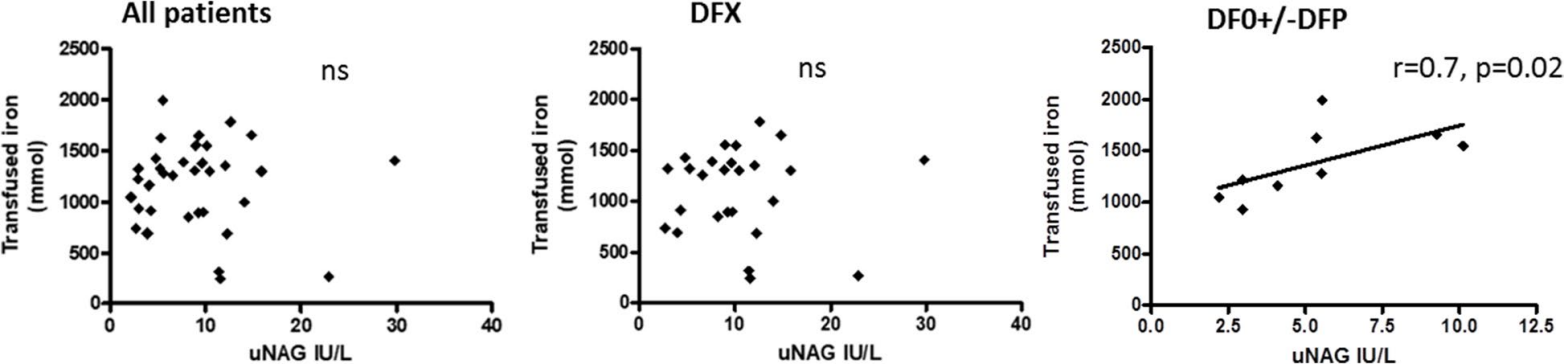
Correlation between uNAG and the amount of iron transfused in prior 10 years in All patients (no correlation found, r = 0.08521, *p* = 0.6), patients treated with DFX (no correlation found, r = 0.023, *P* = 0.8949) and in patients treated with DFO+/−DFP – significant positive (r = 0.7, *P* = 0.02). (DFX) Deferasirox. (DFO) Desferrioxamine. (DFP) Deferiprone

**Fig. 3:**
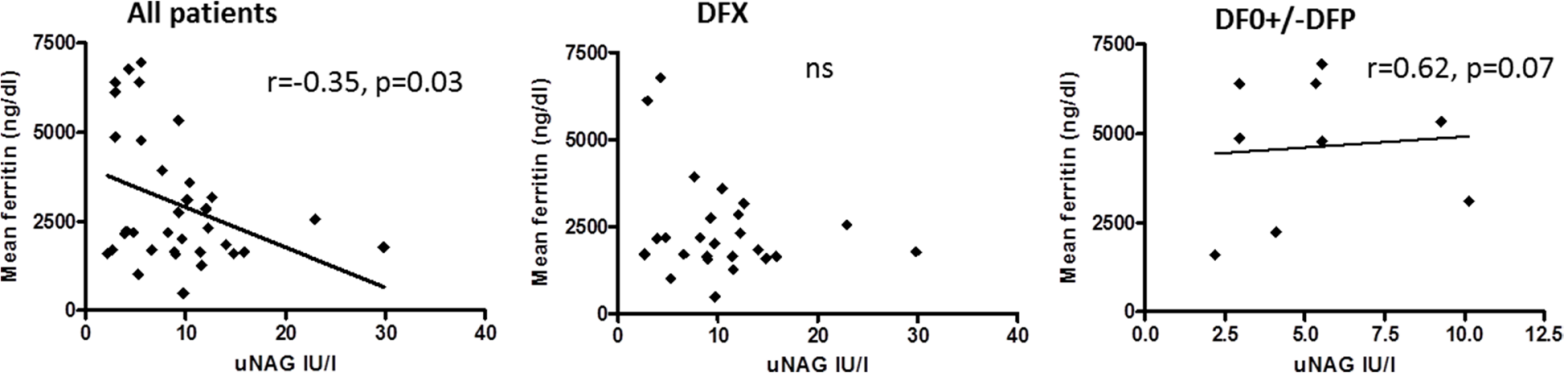
Correlation between uNAG levels and mean serum ferritin for the prior 10 years for All patients (r = − 0.35, *p* = 0.03), the DFX group (r = − 0.028, *p* = 0.88) and the DFO+/−DFP group, significant positive (r = 0.62, *p* = 0.07). (DFX) Deferasirox. (DFO) Desferrioxamine. (DFP) Deferiprone

**Fig. 4:**
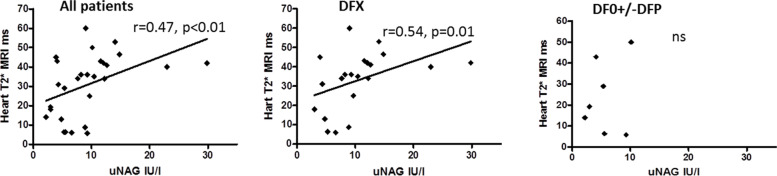
Correlation between uNAG and heart T2* MRI values. Positive correlation in All patients group (r = 0.47, *p* < 0.01). Positive correlation in patients treated with DFX (r = 0.54*. p* = 0.01). No correlation was found in patients treated with DFO+/−DFP (r = 0.2517; *p* = 0.4). Note that lower values for heart T2*MRI indicate worse cardiac function and increased cardiac iron content. (DFX) Deferasirox. (DFO) Desferrioxamine. (DFP) Deferiprone

In patients treated with DFO +/− DFP a significant positive correlation was found between uNAG levels and the 10-year amount of transfused iron (Fig. 2) and between uNAG and mean serum ferritin calculated for the prior 10 years (Fig. 3). This correlation was not found in the group of patients treated with DFX (Fig. 2) and (Fig. 3). A non-significant negative correlation was found between uNAG levels and mean serum ferritin calculated for the prior 10 years for the whole group of patients and in patients treated with DFX (r = − 0.35, *p* = 0.03 and r = − 0.028, *p* = 0.88 respectively) (Fig. 3). In the whole group of patients and in patients treated with DFX a negative correlation was found between uNAG and heart iron load content estimated by T2*MRI (r = 0.47, *P* < 0.01 and r = 0.54. *P* = 0.01 respectively) (Fig. 4). No correlation between uNAG and heart iron load content was found in patients treated with DFO+/−DFP (r = 0.2517; *P* = 0.4).

No correlation was found between uNAG and liver iron load content estimated by T2*MRI**.**

**Fig. 5:**
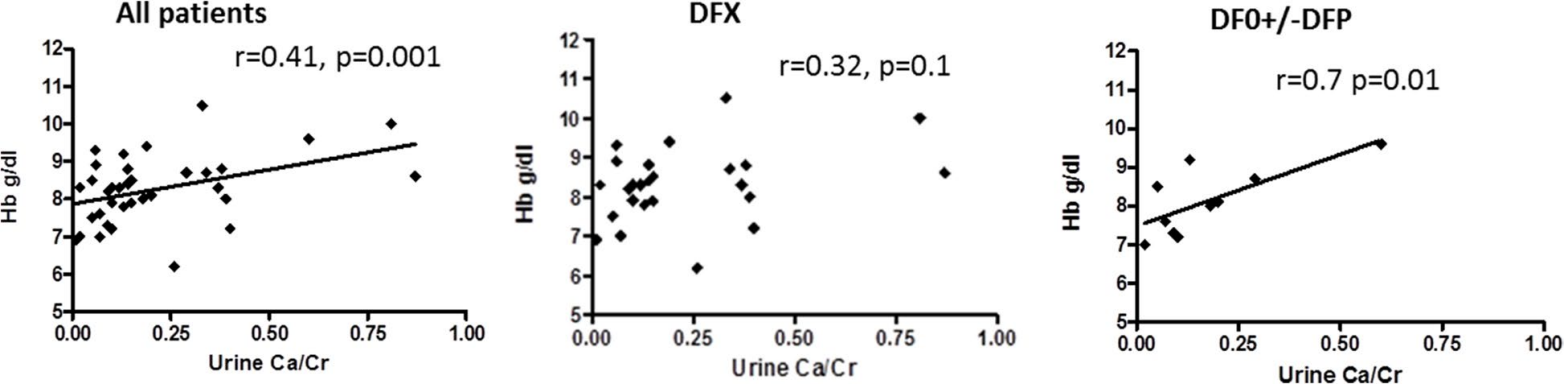
Correlation between urinary Ca/Cr ratio and Hb levels in All patients group (r = 0.41, *p* = 0.001), in patients treated with DFX (r = 0.32, *p* = 0.1) and in patients treated with DFO+/−DFP (r = 0.7, *p* = 0.018). (DFX) Deferasirox. (DFO) Desferrioxamine. (DFP) Deferiprone

A positive correlation was found for urinary Ca/Cr ratio and Hb levels, and was more pronounced in the group of patients treated with DFO+/−DFP than in those treated with DFX (r = 0.7, *P* = 0.018 vs r = 0.41, *P* = 0.001 vs.) (Fig. 5).

No correlation was found between renal tubular parameters and patient age, with the exception of a negative correlation with TmP/GFR (r = − 0.334, *P* = 0.046). The correlation results are summarized in Table 3.

**Table 3:** Correlation between renal parameters and iron status parameters in patients with β-Thalassemia Major and their subgroups according to the chelation treatment, in the current study

Parameters Correlated	Study subgroups (chelation treatment)		
Chelator	DFX *n* = 26	DFO +/−DFP *n* = 10	Total *n* = 36
uNAG vs. Total amount of total iron transfused for prior 10 years	r = 0.02, *p* = 0.89	r = 0.7, *p* = **0.02**	r = 0.08, *p* = 0.6
uNAG vs. Mean serum ferritin for the prior 10 years	r = − 0.028, *p* = 0.88	r = 0.62, *p* = **0.07**	r = − 0.35, *p* = 0.03
uNAG vs. Heart T2* MRI ms.	r = 0.54, *p* = **0.01**	r = 0.2517, *p* = 0.4	r = 0.47, *p* **< 0.01**
Urinary Ca/Cr ratio vs Hb levels	r = 0.32, *p* = 0.1	r = 0.7, *p* = **0.018**	r = 0.41, *p* = **0.001**
Urinary Ca/Cr ratio vs. Mean serum ferritin for the prior 10 year	r = − 0.35, *p* = 0.03	r = − 0.68, *p* = **0.03**	r = − 0.35, *p* = 0.03
Urinary Ca/Cr ratio vs. Amount of iron transfused over the prior10 years	r = −0.12, *p* = 0.5	r = − 0.55, *p* = **− 0.09**	r = − 0.4, *p* = **− 0.014**

### Renal function parameters, what can we learn after ten years after comparing the results of those two studies?

**Fig. 6:**
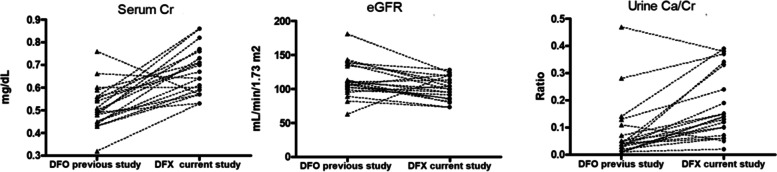
Comparison of serum Cr, eGFR and urine Ca/Cr in the patients reported in the previous study to the same patients [20] treated with DFO in our previous study and with DFX in the current study, Serum Cr (*p* < 0.0001), eGFR (*p* = 0.0093), urinary Ca/Cr (*p* = 0.001) (Wilcoxon matched-pair *t* test). (DFX) Deferasirox. (DFO) Desferrioxamine. (DFP) Deferiprone

Twenty nine patients in the current study were evaluated in our previous study [6]. Comparing the results, we found a significant increase in serum Cr as compared to the previous study in the patients switched with DFX (20 pts), but not in patients who continued DFO or added DFP (9 pts), (*n* = 20, mean 0.51 ± 0.9 vs. 0.67 ± 0.1, *p* = 0.0008). The eGFR was significantly lower as compared to the previous study for patients switched to DFX, but not those treated with DFO+/−DFP, (n = 20, mean GFR first study 113.5 ± 26 vs. 100.1 ± 17, *p* = 0.0093) (Fig. 6).

In the current study for the whole group of patients the urine Ca/Cr ratio increased significantly when compared to the previous study; (mean 0.09 ± 0.11 vs. 0.21 ± 0.2, *p* = 0.001) (Fig. 6) and the percentage of patients with increased Urine Ca/Cr increased from 6.8% in the previous study to 27.7% in the current study, without any difference between treatment groups.

## Discussion

Renal injury in TDT patients is being observed more frequently with prolonged patient survival, and has recently become the object of extensive research [3–5]. Since chronic anemia, iron overload and ICT coexist in virtually all patients, it is hard to isolate and study a specific contributing factor. Our study aimed at examining the tubular and glomerular functions of TDT patients treated with two different ICT; we then compared our results to those of the same patients examined 10 years earlier [6]. We evaluated 36 TDT patients; all of them had normal Cr, serum electrolytes and eGFR.

### Tubular injury

Hypercalciuria was found in 28% of the patients, increased Fe K in 33%, high UAE in 64%, and high levels of TmP/GFR in 25%. Those results are in accordance with previous reports [5, 7]. No significant difference was found between the two ICT groups in those parameters.

**Fig. 7:**
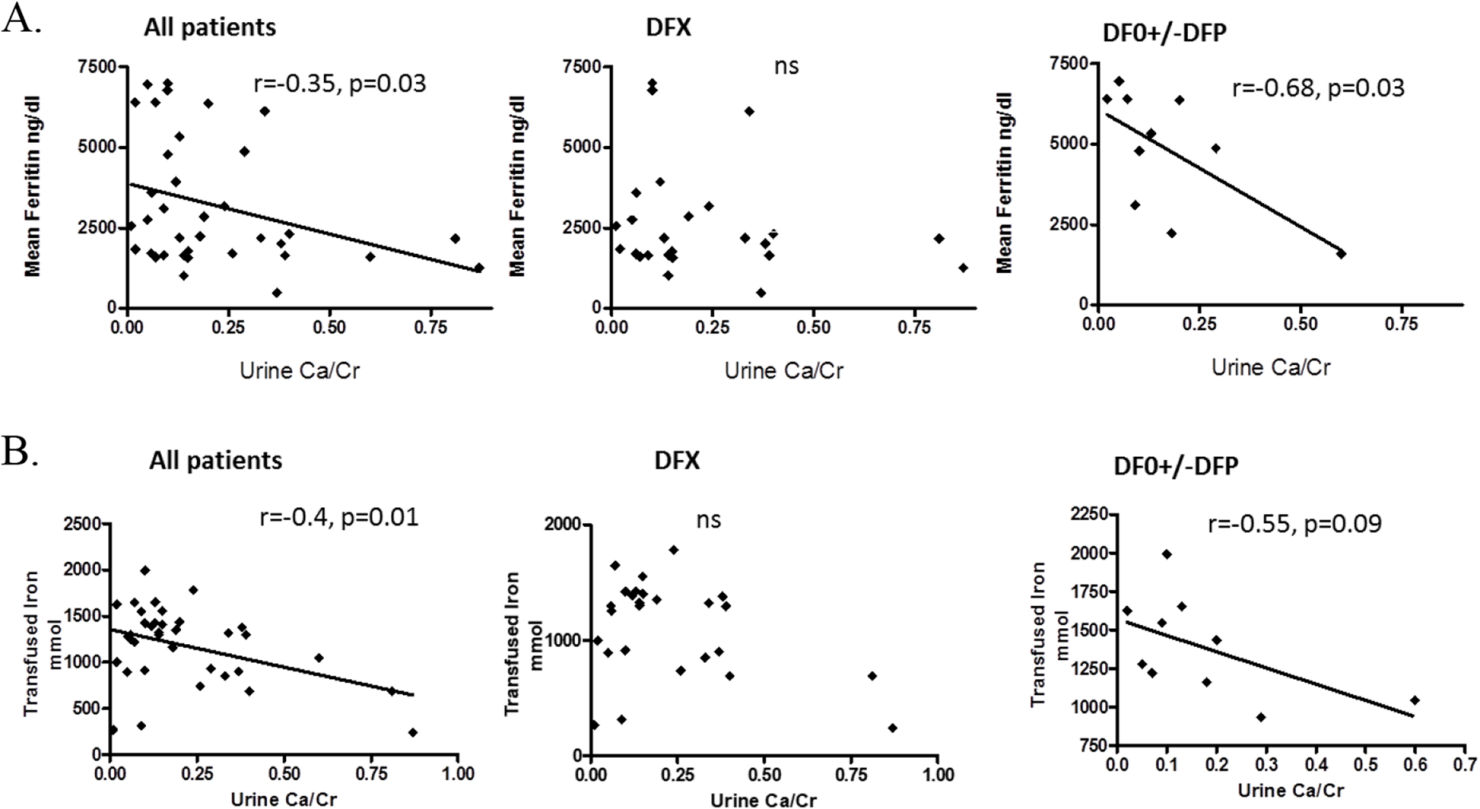
Correlations between urinary Ca/Cr ratio (**A**) in All patients group (r = − 0.35, *p* = 0.03), in DFX group (r = − 0.35, *p* = 0.03) and in the DFO+/−DFP group showing a significant negative correlation (r = − 0.68, *p* = 0.03). Correlation between urinary Ca/Cr ratio and amount of iron transfused over the prior10 years in (**B**) all patients group (r = − 0.4 *p* = 0.014), in DFX group (r = − 0.12, *p* = 0.5) and DFO+/−DFP group showing a significant negative correlation (r = − 0.55 *p* = 0.09). (DFX) Deferasirox. (DFO) Desferrioxamine. (DFP) Deferiprone

Hypercalciuria is a major concern in TDT patients because of its association with osteoporosis [39] and renal stones [40, 41]. In our study, hypercalciuria was significantly negatively correlated with mean serum ferritin for the prior 10 years, and with the 10-year amount of transfused iron (Fig. 7 A, B). Those correlations were stronger in the group of patients treated with DFO+/−DFP. While the frequency of hypercalciuria in our patients was similar to that in Quinn et al. [42], in his study, unlike ours, it was associated with a higher intensity of transfusion. This discrepancy might be explained by the inclusion of non-transfusion-dependent patients in Quinn et al.’s study, and the use of different ICT. Wong et al. [35] reported hypercalciuria in 90% of TDT patients, correlated to DFX dose. Hypercalciuria in our cohort was much less common, but the negative correlation with ferritin level might have been a consequence of the ICT treatment or over chelation. The percentage of patients with hypercalciuria increased significantly in the present study, especially for patients in the DFX group. We do not know if this increment is related to the change to DFX as the principal ICT used by the patients in our study or a tubular damage over the years.

A positive correlation was found between urinary Ca/Cr ratio and Hb levels, and it was stronger in the group of patients treated with DFO+/−DFP. Further studies are needed to clearly define the factors associated with hypercalciuria and groups at risk, who might require a stricter bone density scan program.

Abnormally high uNAG levels, reflecting tubular endothelial injury, were significantly more prevalent and higher in the DFX group compared to the DFO+/−DFP group. Our results are in accordance with prior reports of DFX associated renal tubular dysfunction [6, 29, 32, 33, 43]. Recently Annayev and Karakas [44] reported a higher prevalence of tubular injury, expressed in higher 2-microglobulin levels, in patients treated with a high dose of DFX. A negative correlation (r = − 0.35, *p* = 0.03) was found between uNAG levels and the mean serum ferritin for the prior 10 years. This might also be attributed to over chelation, direct tubular iron toxicity, direct toxic effect of DFX or the cumulative dose of DFX, similar to the trend described by Wong et al. [35] in DFX dose related hypercalciuria.

In patients treated with DFO+/−DFP, a strong positive correlation was found between uNAG levels and the 10-year amount of transfused iron. Such a correlation was not found in patients treated with DFX. Iron renal toxicity has been well described in TDT patients, and Koliakos et al. [23] reported a similar correlation between ferritin levels and uNAG in DFO-treated patients. Michelakakis et al. [25] also reported similar findings, and found that the effect can be reversible in patients well-chelated with DFO.

Heart and liver T2* MRI studies are becoming the standard of care in evaluating tissue iron load in TDT patients, almost completely replacing liver biopsy [45]. In our study, uNAG levels were negatively associated with heart iron content as evaluated by heart T2* MRI; such a correlation was not found with liver iron content. Several previous studies have reported a lack of correlation between heart and liver iron contents [46, 47]. DFX and DFP’s efficiency at reducing heart iron content has been demonstrated [31, 46]. In light of this, our results can be explained by chelation toxicity, with patients who are better chelated as determined by low heart iron content having higher ICT-related tubulopathies. This observation correlates well with our results of a negative correlation between uNAG and ferritin levels.

As already noted, the complex interplay between the factors contributing to renal tubular injury is difficult to untangle, but there appears to be two different trends in the different ICT groups. In the DFO+/−DFP groups, tubular injury seems to be related to iron toxicity or related to the DFX treatment. The use of DFX over years can be the dominant factor in kidney injury. Long term continuous renal function follow up in patients treated continuously with the same chelator can resolve this question. Since usually patients received different chelators during their lives, this comparison seems difficult to perform. In addition, correlations differed in terms of uNAG levels and hypercalciuria, possibly due to the different injury localization inflicted on the renal tubule, and inconsistency in the degree of injury as reflected by iron overload markers, ferritin levels, and heart MRI; one should therefore make conclusions with caution.

Twenty nine of the patients in the current study were evaluated in our previous study [6]. Serum Cr significantly increased from the previous study in patients treated with DFX but not in patients treated with DFO+/−DFP. The eGFR was significantly lower than in the previous study in patients treated with DFX, but not in patients treated with DFO+/−DFP. These findings are worrisome, especially in children who will likely undergo ICT for life. Although all ICTs have been reported to cause renal injury, DFX has been shown to specifically affect eGFR and increase serum Cr. The increase in serum Cr among patients treated with DFX have been considered transitory in literature [29, 48, 49]. .Chronic hyperfiltration, chelator drugs themselves toxicity, and over-chelation appears to be the major contributors to the decline in glomerular filtration rate observed in our study, this in line with previous reports [4, 5, 14–16]. The interindividual differences of patients may be an additional contributor factor but this is difficult to demonstrate. Our results might suggest the possibility of a progressive renal damage, a suggestion that can be proved just after a long-term prospective study based in larger cohorts.

A ten year follow-up study of renal function in TDT patients treated exclusively with DFO [16] reported a mild decrease in eGFR, with most patients remaining within the normal range, similar to our cohort. Lai et al.^17^ reported a significant decline in eGFR in patients with an existing tubular injury; in our cohort, the decrease in eGFR was significantly more common in the DFX group, (*p* = 0.0093), in agreement with previous studies. However, these finding should be viewed in light of the well-reported hyperfiltration in TDT patients [42] and its effect on the interpretation of eGFR, precluding any definite conclusions; more sensitive tools for estimating GFR are warranted.

The limitations of our study are those inherent to a retrospective study; including a limited and relatively small sample size and a heterogeneous treatment protocols and patient group. Some of those limitations arise from the natural history of patients with TDT including the change of treatment regimens throughout time. However, a prospective study of this magnitude will take a long time to perform, and until such a study is done, our study can be valuable to the clinicians taking care of children and adults with TDT.

In summary, tubular injury is common in TDT patients, with higher uNAG levels in DFX-treated vs. DFO+/−DFP-treated patients. Chronic use of DFX was also associated with a non-significant decrease in eGFR (*P* = 0.06). The differences in some tubular function results in the current study when compared to our previous study can arise from diverse interrelated causes as discusses above. Those entire hypotheses should be corroborated or discarded through a sequential longitudinal study in the same cohort of patients followed up for several years. Larger studies are needed to understand the complex mechanism of glomerular and tubular injury in thalassemia, but awareness and close monitoring of underlying renal dysfunction is warranted, especially in patients treated with DFX.

## Data Availability

The datasets generated and/or analyzed during the current study are not publicly available due to patients’ privacy concerns but are available from the corresponding author on reasonable request.
